# Investigating Prime-Pull Vaccination through a Combination of Parenteral Vaccination and Intranasal Boosting

**DOI:** 10.3390/vaccines8010010

**Published:** 2019-12-31

**Authors:** Carla B. Roces, Maryam T. Hussain, Signe T. Schmidt, Dennis Christensen, Yvonne Perrie

**Affiliations:** 1 Strathclyde Institute of Pharmacy and Biomedical Sciences, University of Strathclyde, Glasgow G4 0RE, UK; carla.roces-rodriguez@strath.ac.uk (C.B.R.); maryam.hussain@strath.ac.uk (M.T.H.); 2 Center for Vaccine Research, Statens Serum Institut, 2300 Copenhagen, Denmark; sxs@ssi.dk (S.T.S.); den@ssi.dk (D.C.)

**Keywords:** PLGA, adjuvants, prime-pull, tuberculosis, powder, lungs

## Abstract

Formulation of inhalable delivery systems containing tuberculosis (TB) antigens to target the site of infection (lungs) have been considered for the development of subunit vaccines. Inert delivery systems such as poly (lactic-co-glycolic acid) (PLGA) are an interesting approach due to its approval for human use. However, PLGA suffers hydrolytic degradation when stored in a liquid environment for prolonged time. Therefore, in this study, nano- and microparticles composed of different PLGA copolymers (50:50, 75:25 and 85:15), sucrose (10% *w*/*v*) and L-leucine (1% *w*/*v*) encapsulating H56 TB vaccine candidate were produced as dried powders. In vitro studies in three macrophage cell lines (MH-S, RAW264.7 and THP-1) showed the ability of these cells to take up the formulated PLGA:H56 particles and process the antigen. An in vivo prime-pull immunisation approach consisting of priming with CAF01:H56 (2 × subcutaneous (s.c.) injection) followed by a mucosal boost with PLGA:H56 (intranasal (i.n.) administration) demonstrated the retention of the immunogenicity of the antigen encapsulated within the lyophilised PLGA delivery system, although no enhancing effect could be observed compared to the administration of antigen alone as a boost. The work here could provide the foundations for the scale independent manufacture of polymer delivery systems encapsulating antigens for inhalation/aerolisation to the lungs.

## 1. Introduction

Tuberculosis (TB) remains a major health problem, affecting approximately a quarter of the world’s population. The current TB vaccine, the Bacillus Calmette–Guérin (BCG) vaccine, was discovered 1921 and it is intradermally administrated in children at the time of birth. BCG is only partially effective in protecting children against disseminate TB, showing severely variable protection versus primary infection in children and it does not protect against pulmonary TB, which is the most common form of TB [1]. *Mycobacterium tuberculosis* (Mtb) bacilli spread through the air from one person to another resulting in infection within the lungs. Alveolar macrophages (AM) are the first line of defence for the clearance of inhaled pathogens. However, AMs are the TB bacilli host site for the infection; therefore, this impairs the ability of these macrophages to promote protection. Formulation of inhalable delivery systems containing TB antigens to target the infection site could be an approach for the development of a subunit vaccine against this disease. Nano- and microparticles formulated using materials approved by the Food and Drug Administration and the European Medicines Agency for human use (e.g., Poly-lactic glycolide aka PLGA) have been extensively investigated for pulmonary delivery due to their high safety profile. Due to their hydrolytic degradation in aqueous suspension, techniques such as freeze-drying are used to overcome this problem, and thus, increasing the stability of these particles. Lyophilisation also allows for the engineering of particles with suitable aerodynamic parameters to achieve deposition in the appropriate lung region, since particle size is one of the main factors dictating the aerosol deposition within the airways. Particles larger than 5 µm will be most likely deposited in the upper airways due to impaction whereas particles from 0.5 µm and 5 µm will be deposited due to sedimentation in the lower airways. Particles smaller than 0.5 µm, due to random Brownian motion, will be most likely deposited in the alveoli by diffusion; the main drawback is that due to the small particle size, these particles might be exhaled. Thus, an optimum particle size for pulmonary delivery with deposition in the lower airways would be between 0.5 and 5 µm.

It is believed that the ineffectiveness of BCG protecting against pulmonary TB is due to the absence of antigen-specific T cells in the lungs [2]. Although the mechanism underlying TB protection is not well understood, the majority of the studies agree on the critical protective role that T cell mediated immunity plays against TB [3,4,5]. Recruitment of T cells to the infection site (lungs) takes approximately 10 days after natural infection, as Mtb infection lessens the T cell migration to the lungs. Overcoming this delay is crucial for the design and development of a successful TB vaccine since this delay allows Mtb bacilli to proliferate in the AMs. Thus, respiratory mucosal vaccines promoting the recruitment of T cells in the lungs in order to control Mtb growth might be a promising strategy against pulmonary TB, as it has been shown to be critical for early infection control [6].

Therefore, within this study, we have evaluated an immunisation strategy based on the parenteral priming of a T cell helper (Th) −17 response followed by mucosal boosting using free antigen or aerodynamically engineering particles. The cationic adjuvant formulation (CAF) 01 was selected as the “primer” since it has been previously reported that priming with CAF01 + Antigen facilitates a mixed Th1/Th17 response promoting rapid T cell homing to the lungs after airway boosting [7] and that the administration of CAF01 in combination with H56 TB antigen by parenteral route produces early CD4 T cell responses in the lungs after TB infection [5]. PLGA was selected as the delivery system for the intranasal “booster” immunisation due to its high safety profile and documented efficacy for pulmonary delivery [8]. Therefore, we have engineered dry powders for inhalation containing PLGA as delivery system for the delivery of the H56 antigen into the deep lungs. For this purpose, the choice of cryoprotectant was evaluated as well as the aerodynamic diameter and particle deposition of the powders within the lungs using a Pharmacopoeia approved airway simulator; Morphological microscopy for the characterisation of the dry powders and in vitro viability, uptake and antigen processing in three macrophage cell lines (MH-S, RAW264.7 and THP-1). Selected PLGA nano- and microparticles were further evaluated as part of a “prime-pull” immunisation protocol where mice were primed with CAF01:H56 (2 × s.c.) and subsequently boosted intranasally with PLGA:H56 particles.

## 2. Materials and Methods

### 2.1. Materials

For the preparation of delivery systems, poly (lactic-co-glycolic acid) 85:15 (Mw: 50,000–75,000), 75:25 (Mw: 66,000–107,000), 50:50 (Mw: 30,000–60,000), sodium hydroxide (NaOH), ovalbumin (OVA), sucrose, L-leucine and the stabiliser polyvinyl alcohol (PVA Mw: 31,000) were purchased from Sigma-Aldrich Company Ltd., Poole, UK. DQ-ovalbumin™ and DiI Stain (1,1′-Dioctadecyl-3,3,3′,3′-Tetramethylindocarbocyanine Perchlorate) were purchased from ThermoFisher Scientific (Loughborough, UK). 2-amino-2-(hydroxymethyl)-1,3-propanediol (Tris) was obtained from ICN Biomedicals Inc. (Aurora, OH, USA) and prepared at a 10 mM concentration and pH 7.4 unless otherwise stated. All other reagents were of analytical grade and purchased from commercial suppliers. The tuberculosis vaccine candidate H56 was donated by Statens Serum Institut (SSI), Copenhagen, Denmark.

### 2.2. Preparation of Freeze-Dried PLGA:H56 Nanoparticles and PLGA:H56 Microparticles

PLGA (50:50, 75:25 and 85:15) nanoparticles encapsulating H56 TB antigen were manufactured using microfluidics (Nanoassemblr^®^ Benchtop, Precision Nanosystems Inc., Vancouver, BC, Canada) as described previously (Roces et al., 2020 accepted manuscript). Particles were produced at a flow rate of 10 mL/min, 1:1 aqueous: solvent ratio. Final polymer and antigen concentration were 5 mg/mL and 0.25 mg/mL in Tris buffer. On the other hand, PLGA (50:50, 75:25 and 85:15) microparticles were prepared using the double emulsion solvent evaporation method. Briefly, 20 µL of antigen (10 mg/mL stock) were added to 417 µL of 3% *w*/*v* PLGA in chloroform and vortexed for 1.5 min to form the primary emulsion. Subsequently, the emulsion was mixed with 10 mL of PVA (10% *w*/*v* in dH_2_O) at 8000 rpm for 3 min (Homogenizer Ultraturrax T25, IKA, Oxford Business Park, Oxford, UK). Solvent was evaporated overnight and microparticles were then three times centrifuged (Hermle Z323K, Labnet International Inc., Edison, NJ, USA) for 20 min at 5500× *g*, and washed each time with 10 mL of dH_2_O. After the last wash, pellet was resuspended with buffer (Figure S1).

Specific amounts of cryoprotectants (10% sucrose and 1% L-leucine (*w*/*v*)) were added into both, nano- and microparticles post-manufacture. Particles were then frozen for a minimum of 2 h at −80 °C before freeze-drying (main drying −50 °C for 18 h and final drying −20 °C for 6 h) [9,10]. After lyophilisation, particles were re-suspended in ultrapure water. Quantification of the antigen encapsulated within the PLGA particles was performed by reverse phase HPLC using a UV detector following the method described by Roces et al., 2020 (accepted manuscript).

### 2.3. Particle Size and Zeta Potential of PLGA Nano- and Microparticles

The particle size, polydispersity (PDI) and zeta potential of the PLGA nanoparticles was determined using a Malvern nano ZS (Malvern PANalytical, Worcestershire, UK). Samples were previously diluted in filtered Tris buffer (10 mM, pH 7.4) to obtain a final concentration of 0.25 mg/mL in the cuvette (1/20 dilution). Malvern Dispersion Technology Software (DTS) v.7.12 was used for data analysis and collection. The particle size and size distribution (SPAN) of the PLGA microparticles was determined by laser diffraction analysis using a Mastersizer 2000 (Malvern PANalytical, Worcestershire, UK). Samples were dispersed in water into the sample dispersion cell unit while stirring at 2000 rpm until above 10% obscuration was obtained. PLGA refractive index was set at 1.43 and three measurements of each sample were recorded every 12 s. Scanning electron microscopy (SEM) was used for the morphological particle analysis and to compare the particle size and size distribution of the nano- and microparticles. This procedure was carried out externally by David McCarthy (DMmicroscopy). Images were taken on a FEI Quanta FEG, Eindhoven, The Netherlands. The voltage used is shown at the foot of each image, usually 5 or 8 KV.

### 2.4. Pharmacopoeia Airway Simulator: Next Generation Impactor (NGI)

The in vitro aerosol dispersion performance of the antigen loaded PLGA powders was determined using an NGI (NGI™; Copley Scientific Ltd., Nottingham, UK) equipped with vacuum pump (Model HCP5) and a critical flow controller (TPK 2000). Dry powders (10, 20, 30 or 50 mg) were filled into a size 3 hydroxypropyl methylcellulose capsule, and inserted into the aeroliser (Plastiape Monodose Dry Powder Inhaler, Plastiape S.p.a, Milan, Italy). The capsules were then punctured and the aeroliser attached to the induction port through a silicone mouthpiece adaptor. Each capsule was aerosolised at 60 L/min flow rate (verified using a digital flow meter) for 4 s (temperature = 23 °C; Pa = 101.6 kPa; Relative Humidity = 41.7%). After each shot, the powders deposited in the induction port and in all the stages were collected by rinsing with ultrapure water and analysed as percentage deposition of the powders using a bicinchoninic acid (BCA) assay (Pierce™ BCA Protein Assay Kit, Sigma Aldrich, Poole, UK). For the calculation of the aerosol performance of the powders, the following equations were followed:

$$\mathrm{Fine}\;\mathrm{particle}\;\mathrm{dose}\;\left(\mathrm{FPD}\right)\;=\;\mathrm{mass}\;\mathrm{of}\;\mathrm{particles}\;\mathrm{on}\;\mathrm{Stages}\;2\;\mathrm{through}\;7\;\mathrm{Fine}\;\mathrm{particle}\;\mathrm{fraction}\;\left(\mathrm{FPF}\right)=\frac{\mathrm{Fine}\;\mathrm{particle}\;\mathrm{dose}}{\mathrm{Initial}\;\mathrm{particle}\;\mathrm{mass}\;\mathrm{loaded}\;\mathrm{into}\;\mathrm{the}\;\mathrm{capsules}}*100\%\;\mathrm{Respirable}\;\mathrm{fraction}\;\left(\mathrm{RF}\right)=\frac{\mathrm{Mass}\;\mathrm{of}\;\mathrm{particles}\;\mathrm{on}\;\mathrm{stages}\;2\;\mathrm{to}\;7}{\mathrm{Total}\;\mathrm{particle}\;\mathrm{mass}\;\mathrm{on}\;\mathrm{all}\;\mathrm{stages}}*100\%\;\mathrm{Emitted}\;\mathrm{dose}\;\left(\mathrm{ED}\right)=\frac{\mathrm{Initial}\;\mathrm{mass}\;\mathrm{in}\;\mathrm{capsules}-\mathrm{final}\;\mathrm{mass}\;\mathrm{remaining}\;\mathrm{in}\;\mathrm{capsules}}{\mathrm{Initial}\;\mathrm{mass}\;\mathrm{in}\;\mathrm{capsules}}*100\%$$

The mass median aerodynamic diameter (MMAD) was calculated from plotting the logarithm of the cut-off diameters against the cumulative mass percentage calculated from each stage. From that graph, the MMAD was determined as the point where 50% of particle deposition crossed the x-axis.

### 2.5. In Vitro Cell Viability, Particle Uptake and Antigen Processing in Three Macrophages Cell Lines: THP-1, MH-S and RAW264.7

The toxicity of the PLGA nano- and microparticles was examined using cell titre blue (CTB) assay and the amount of cells alive was quantified using a spectrophotometer at 590 nm wavelength. Briefly, confluent cells were plated on a 96 well plate at a density of 1–2 × 10^6^/mL. PLGA nano- and microparticles were added 24 h later at a concentration range from 6–200 µg/mL. Calculation of cell viability was based on the difference between the positive control (untreated cells) and the sample test (cells exposed to PLGA particles).

PLGA nano- and microparticles were fluorescently labelled using Dil stain during manufacturing process (0.5% *v*/*v* Dil into polymer dissolved in organic solvent) for the quantification of the particle uptake. Samples were then diluted down to 20 µg/mL in serum free RPMI 1640 medium and added at 1:1 *v*/*v* ratio to the cells, which were plated at a density of 1 × 10^5^ cells/mL in 24 well plates. This mixture was incubated at 37 °C 5% CO_2_ and at specific intervals (0.5, 1, 2 and 3 h) 200 µL of co-culture was mixed with 200 µL of ice-cold cRPMI before analysis. The interaction of PLGA particles with the cells was analysed using flow cytometry (minimum of 5000 events per sample). As a negative control, same experiments but at 4 °C was carried out to stop endocytosis.

Cell antigen processing was quantified using the DQ™ ovalbumin (self-quenched conjugate) which fluoresces when it undergoes enzymatic degradation by the cells. Thus, PLGA nano- and microparticles encapsulating a mixture (1:1 *w*/*w*) of OVA and DQ-OVA were produced by either microfluidics or the double emulsion method and same protocol as for the particle uptake was followed.

### 2.6. Prime-Pull Immunisation Protocol

**Figure 1:**
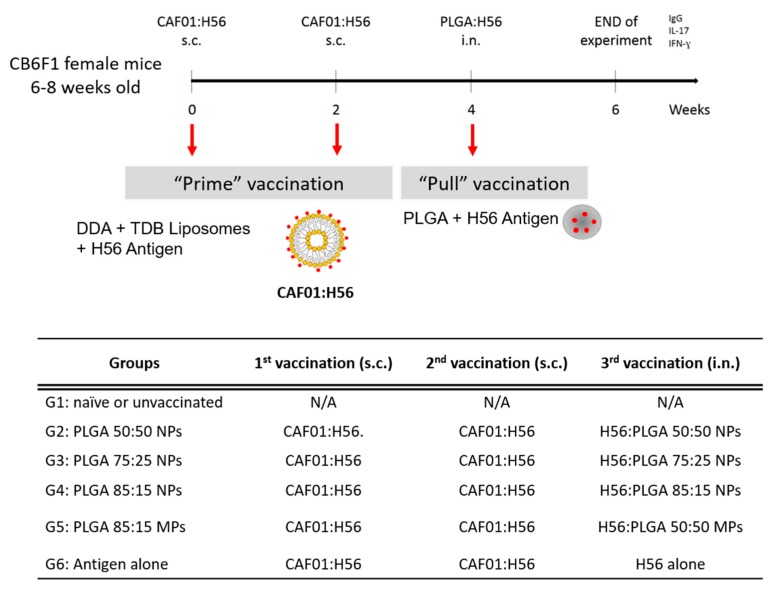
Immunisation protocol followed for the prime-pull immunisation of CB6F1 mice to investigate the production of antigen specific antibodies and cytokines for the development of a vaccine effective against pulmonary tuberculosis (TB). Mice were immunised three times (2 × subcutaneous (s.c.) with CAF01:H56 and 1 × intranasally (i.n.) with PLGA:H56) with 2 weeks interval between immunisations. 2 weeks after the intranasal boosting, mice were terminated and data analysed. The table shows the vaccines administered to the CB6F1 female mice for the immunisation study following a prime-pull protocol. All mice were parenterally primed twice with CAF01:H56 except for the unvaccinated group. Each group was boosted with the same H56 antigen dose encapsulated in either poly (lactic-co-glycolic acid) (PLGA) nanoparticles (NPs) (50:50, 75:25 and 85:15) or PLGA 85:15 microparticles (MPs).

All animal experiments were conducted at Statens Serum Institut in accordance with regulations of the Danish Ministry of Justice and animal protection committees by Danish Animal Experiments Inspectorate Permit 2009/561-1655, 2012-15-2934-00272, 2014-15-2934-01065 and in compliance with EU Directive 2010/63. Six groups of 6 female (6–8 weeks of age) Balb/c × C57BL/6 crossed mice (CB6F1 mice) were immunised three times with 2 week intervals between each immunisation. Mice were primed twice with CAF01:H56 subcutaneously (DDAB:TDB:H56 250/50/5 µg dose in a total volume of 200 µL) and boosted intranasally with PLGA:H56 nano- and microparticles (10 µg H56 per 40 µL dose split over 20 µL per nostril). Two weeks after the final immunization, mice were terminated and blood and organs isolated. CAF01:H56 was prepared at Statens Serum Institut, whereas the PLGA boosters were prepared at the University of Strathclyde (Figure 1).

#### 2.6.1. Organ Processing

Mice were intravenously injected with 250 µL anti-CD45.2- Fluorescein isothiocynate (FITC) labelled antibodies (2.5 µg in 250 µL PBS) and terminated 3 min after injection under CO_2_ atmosphere and individual organs isolated. Individual spleen and lungs were isolated and were kept ice-cold until further processing. Lymph nodes (mediastinal and tracheobronchial) were isolated and pooled together. Blood samples were withdrawn for antibody analysis. Spleens and lungs from each mouse were isolated and processed. Cells were counted and were re-stimulated with either 100 µL ConA (5 µg/mL), RPMI media or H56 (5 µg/mL) and incubated at 37 °C, 5% CO_2_ and 95% humidity for 72 h. Subsequently supernatants were harvested and stored at −20 °C for further processing. Total IgG antibody quantification was studied in the serum and in the supernatants from the lungs using direct ELISA. Results are plot as the Log10 of the dilution against the measured optical density value (OD_450_). The production of cytokines IL-17 and IFN-γ was analysed in supernatants from re-stimulated splenocytes and lung lymphocytes following a sandwich ELISA.

#### 2.6.2. Intracellular Fluorescence Activated Cell Sorting Staining (icFACS)

Antigen specific T cell production of both cytokines, IFN-γ and IL-17, was measured by intracellular flow cytometry. Mice were intravenously administered with fluorescein isothiocyanate (FITC)-labelled anti-CD45 monoclonal antibody (iv.CD45) CD45.2-FITC prior to euthanization, which results in staining lymphocytes in the blood and therefore differentiating between blood cells (vasculature) from cells in the lung parenchyma [11]. Spleens, lymph nodes (mediastinal and tracheobronchial) and lungs were stimulated with H56 antigen. The panel used for icFACS was surface stained for CD4 (CD4-APC-eFluor780), CD44 (CD44-PE) and intracellular stained for IL-17 and IFN-γ (IFNg-PE-Cy7 and IL-17-PerCp-Cy5.5). Compensation beads were used instead of cells. Results are expressed as percentage of activated (CD44+) cytokine producing (IFN-γ +, IL17+) cells of the total CD4+ T-cell population (Gating strategy shown in Figure S2).

### 2.7. Statistical Analysis

Data presentation, analysis and interpretation was performed at USTRATH using GraphPad Prism 7 software (GraphPad Soft-ware, La Jolla, CA, USA) and Microsoft Excel. Means and standard deviations are plotted on the graphs. Statistical analysis of data was calculated by one- way analysis of variance (ANOVA) and when significant differences were indicated, differences between means were determined by Tukey’s post hoc test. Statistical differences with *p* < 0.05 were considered significant.

## 3. Results

The production of antigen loaded PLGA nanoparticles manufactured using the microfluidics was optimised previously (Roces et al., 2020 accepted manuscript). Particles should have sizes from 500 nm to 5 µm for better deep lung deposition and interaction with AMs [12,13]. Therefore, the microfluidic process parameters selected were 10 mL/min flow rate and 1:1 aqueous:organic ratio, for the production of the largest particle size possible. However, particle sizes obtained by this method are yet not large enough for deposition in the deep lungs (from 150 nm to 500 nm, depending on the PLGA copolymer selected) [14]; thus, further aerodynamic modification of the particles is required. For this reason, and due to the chemical/physical stability of PLGA, these nanoparticles were formulated as dry powders using freeze-drying with prior addition of cryoprotectants to prevent these particles from agglomeration/fusion [15,16].

### 3.1. Effect of Freeze-Drying on the Physicochemical Characteristics of Antigen Loaded PLGA Nano- and Microparticles

**Figure 2:**
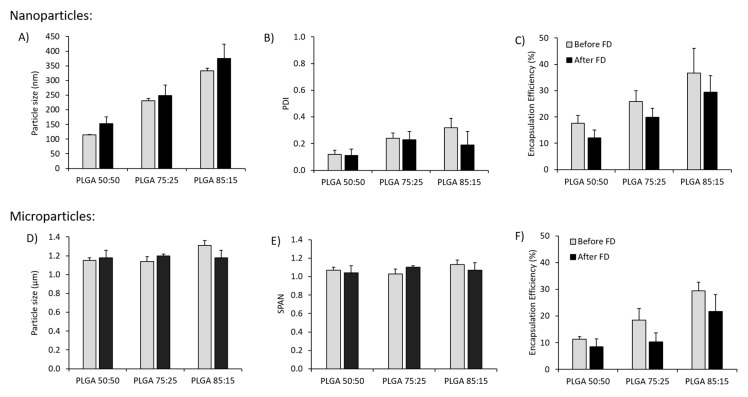
Physicochemical characteristics of the antigen loaded PLGA nanoparticles (microfluidics total flow rate (TFR) 10 mL/min, flow rate ratio (FRR) 1:1) and microparticles (double emulsion method) containing 10% (*w*/*v*) sucrose and 1% (*w*/*v*) L-leucine before freeze drying (FD) and after reconstitution of the dried powders: (**A**,**D**) Particle size, (**B**,**E**) polydispersity index (PDI) or SPAN and (**C**,**F**) Encapsulation efficiency (%) for nanoparticles and microparticles respectively. PDI is defined as the standard deviation of the particle size distribution divided by the mean particle diameter and gives an estimation of the uniformity of the particles. SPAN is also an indication of polydispersity for larger particles. All results represent the mean ± SD of at least three different batches.

PLGA 50:50, 75:25 and 85:15 nanoparticles formulated using microfluidics were freeze-dried and reconstituted with ultrapure water prior to measure their physicochemical characteristics. Sucrose concentrations between 2 and 10% (*w*/*v*), and L-leucine concentrations between 0.5% and 1% (*w*/*v*), either alone or in combination, were tested as lyoprotectants (Figure S3). Based on measured changes in particle size and PDI after reconstitution, the combination of 10% (*w*/*v*) sucrose and 1% (*w*/*v*) L-leucine was selected to prepare antigen loaded nanoparticles and microparticles (Figure 2). After freeze-drying of the antigen loaded nano and microparticles with the combination of 10% sucrose and 1% L-leucine incorporated within, the physicochemical characteristics remained unaffected in terms of particle size (Figure 2A,D), dispersity (Figure 2B,E) and loading (Figure 2C,F).

### 3.2. Morphological Characterisation of the Antigen Loaded PLGA Nano- and Micro-Particles

**Figure 3:**
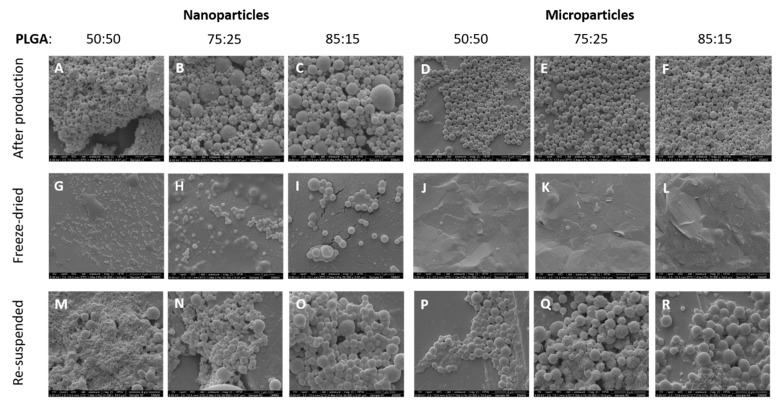
Scanning electron microscope (SEM) images of the antigen loaded PLGA nanoparticles and microparticles with different copolymer ratio (50:50, 75:25 and 85:15), (**A**–**F**) after production, (**G**–**L**) after freeze-drying (powder state) and (**M**–**R**) after re-suspension in ultrapure water. The scale-bars shown at the bottom of each image represent 1 micron (**A**–**C**,**H**,**I**,**N**,**O**), 5 microns (**D,E,F**) or 4 microns (**G**,**J**–**M**,**P**–**R**).

Figure 3 shows the micrographs of the antigen loaded PLGA 85:15, 75:25 and 50:50 nanoparticles and microparticles after production, after freeze-drying (powder state) and after reconstitution of the dry powder using ultrapure water. The morphology of the antigen loaded PLGA particles before freeze-drying and after reconstitution was preserved. Interestingly, the surface of the particles produced with different monomer ratios was different. Overall, all three copolymers showed a rough surface but this was more marked for PLGA 75:25. This rough surface, also reported as wrinkle surface in literature, has been related to hydrophobicity and surfactant-like properties of L-leucine, which alters the surface viscosity of the particles [17,18]. Another theory to explain the increased roughness in the particle surface after freeze-drying might be due to the removal of water during the freeze-drying process. This change has been seen by Fonte et al. when investigating the co-encapsulation of cryoprotectants for increasing the stability of insulin-loaded PLGA nanoparticles after freeze-drying [19]. However, PLGA 50:50 microparticles did not display this wrinkle surface. PLGA 75:25 without the addition of L-leucine also exhibited this rough surface; therefore, this change in surface morphology cannot be attributed to the inclusion of L-leucine into the formulation nor the freeze drying method.

In the powder samples, nano- and microparticles can be seen embedded in the cryoprotectant matrix, whereas the reconstituted powders showed spherical particles in the nanometre range (for the particles produced using microfluidics) and in the micrometre range (for the particles produced using the double emulsion method). The inclusion of cryoprotectant in the PLGA formulation creates a continuous glassy matrix embedding the nanoparticles, which has previously been reported in the literature by other groups [19,20,21]. Particle sizes from the SEM micrographs were in agreement with the values obtained from the DLS measurements.

### 3.3. PLGA Nano- and Microparticles Are Deposited in the Deep Lungs

**Figure 4:**
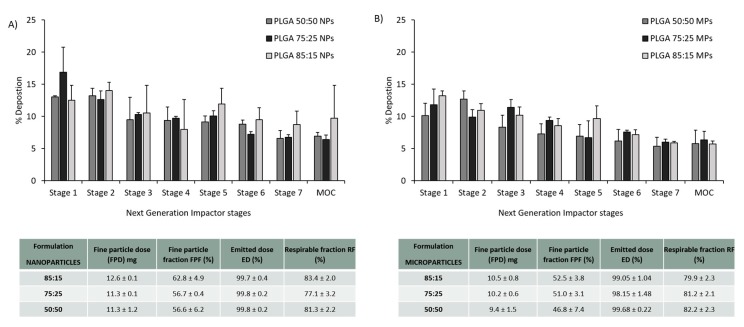
Aerosol dispersion performance of the antigen loaded freeze dried (**A**) PLGA nanoparticles and (**B**) PLGA microparticles with different copolymer ratios 50:50, 75:25 and 85:15, as percentage deposition on each stage of the Next Generation Impactor (NGI). Results represent mean ± SD, n = 3 of independent batches. The table shows the calculated aerolisation parameters of the PLGA powders prepared using freeze drying: fine particle dose (FPD), fine particle fraction (FPF), emitted dose (ED) and respirable fraction (FR), after Next Generation Impactor (NGI).

**Figure 5:**
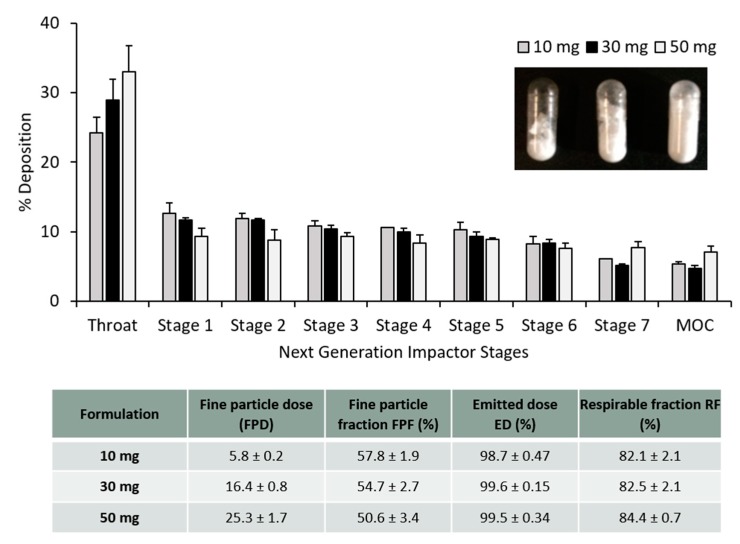
Aerosol dispersion performance of the freeze dried antigen-loaded PLGA 85:15 TFR, 10 FRR 1:1 nanoparticles as percentage deposition on each stage of the Next Generation Impactor (NGI). Results represent mean ± SD, n = 3 of independent batches. Different amounts of dry powder were filled into the size 3 capsule: 10 mg, 30 mg and 50 mg. The table shows the calculated aerolisation parameters of the PLGA powders prepared using freeze drying: fine particle dose (FPD), fine particle fraction (FPF), emitted dose (ED) and respirable fraction (FR), after Next Generation Impactor (NGI).

The particle deposition (%) on each stage of the NGI was estimated and plotted in Figure 4. According to some reports, to achieve good deposition in the deep lungs, particles should have an aerodynamic diameter from 1–2 µm [8]. The calculated MMAD was between 1–1.8 µm for the PLGA nanoparticles whereas for PLGA microparticles the calculated MMAD was between 1.3–2.5 µm. It is not recommended to give more than 10–20 mg of powder in one actuation shot. To do so would trigger a cough reaction in the patient, resulting in the loss of the inhaled antigen into the air. Figure 4 shows the particle deposition of the PLGA nanoparticles and microparticles (capsule filled with 20 mg). In order to screen the amount of powder filled within the capsules for the dry powder inhaler, different amounts of powder (10, 30 and 50 mg) were weighed and filled manually into the capsules as well. Figure 5 shows the particle deposition at the different stages of the NGI when different amounts were added into the capsules. The higher the amount loaded into the capsule, the higher the deposition of the powder in the throat, whereas no significant differences were observed in the deposition of these particles in the deep lungs (stages 5–7). The emitted dose (ED%), which is defined as the amount of powder exiting the capsule compared to the initial amount loaded, was determined by weight after inhalation. The average ED% of the antigen loaded PLGA powders was determined to be 100% independent of the either the copolymer used, the initial amount of powder added into the capsule or the particle production method used. The calculated respirable fraction (RF) as a percentage was above 80% in all the formulations tested. The fine particle fraction (FPF), which is the mass of polymer/antigen reaching stages below 5 µm was approximately 60% for the antigen loaded PLGA nanoparticles and significantly lower (~50%) for the loaded PLGA microparticles.

### 3.4. In Vitro Studies in Murine and Human Origin Cell Lines: Cell Viability, Uptake and Antigen Processing

**Figure 6:**
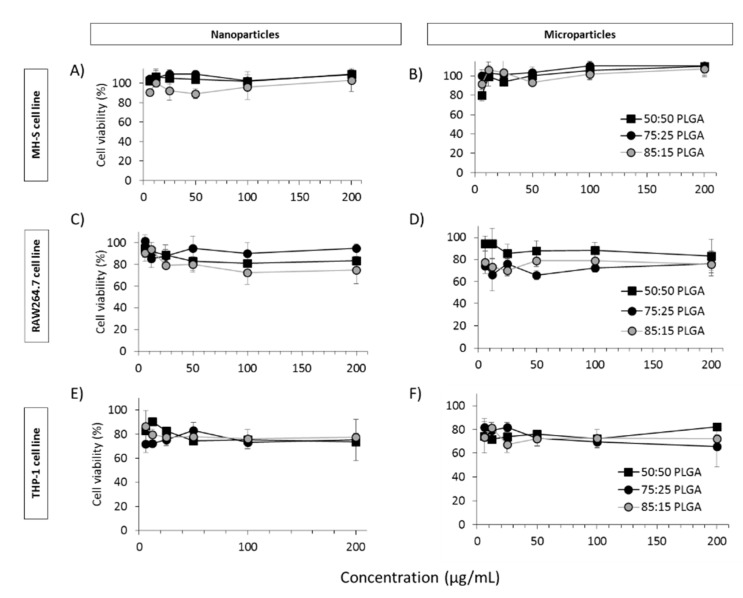
CellTiter-Blue^®^ assay for the quantification of nanoparticle (**A**,**C**,**E**) and microparticle (**B**,**D**,**F**) cell viability in three different cell lines: MH-S (**A**,**B**), RAW264.7 (**C**,**D**) and THP-1 (**E**,**F**).

Three macrophage cell lines were screened for viability, uptake and processing ability when exposed to PLGA nanoparticles and microspheres containing antigen. Two of the cell lines were of mouse origin, RAW264.7 cells (mouse derived monocytes) and MH-S (alveolar macrophages). The other, THP-1 monocytes, was of human origin and was used after stimulation into macrophages. These three cell lines were selected to give us an initial broad screen of cell types. In vitro cytotoxicity studies carried out in both nanoparticles and microparticles (Figure 6) showed no toxic effects on either MH-S, THP-1 or RAW264.7 cells at all concentrations evaluated (6, 12, 25, 50, 100 and 200 µg/mL). In both cases, >75% of cells were viable even at the highest concentration of 200 µg/mL tested.

**Figure 7:**
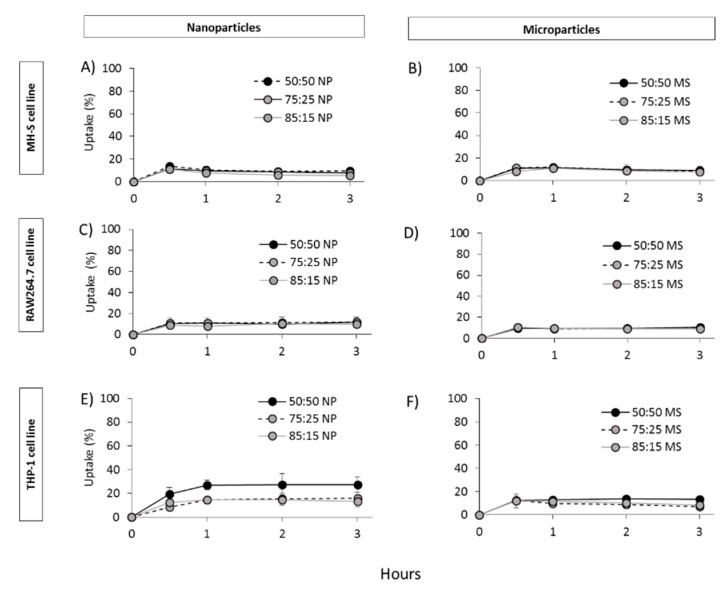
PLGA nanoparticle (**A**,**C**,**E**) and microparticle (**B**,**D**,**F**) uptake by MH-S (**A**,**B**), RAW264.7 (**C**,**D**) and THP-1 (**E**,**F**) cells at 4 °C.

**Figure 8:**
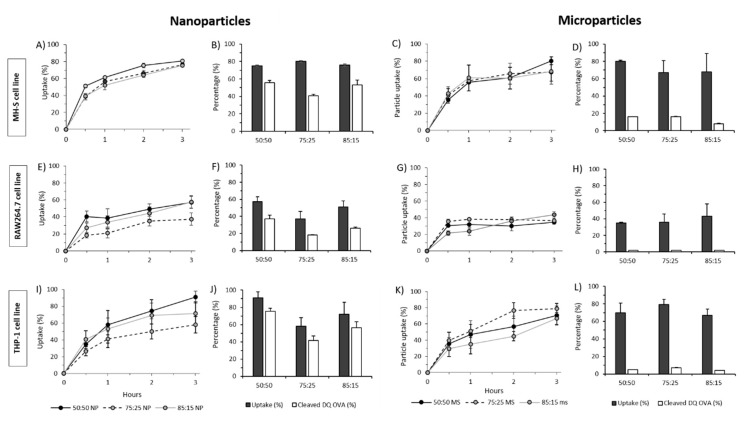
PLGA nanoparticle and microparticle uptake and protein cleaved in MH-S, RAW264.7 and THP-1 at 37 °C. DQ-OVA was encapsulated in PLGA nanoparticles and microparticles and the amount cleaved was calculated as percentage of the initial amount after 48 h exposure to (**A**–**D**) MH-S cells, (**E**–**H**) RAW264.7 and (**I**–**L**) THP-1 cells.

Uptake studies were carried out at 4 °C (Figure 7) and 37 °C (Figure 8) and to determine whether the particles were efficiently taken up by endocytosis or attached to the cell surface since, due to the inhibition of endocytosis at low temperatures (energy dependent process). We observed that particle uptake was significantly lower at 4 °C (Figure 7) compared to 37 °C (Figure 8), suggesting that indeed particle uptake is mediated by endocytosis. In terms of cell uptake, in general, results showed the ability of all three cell lines to take up PLGA particles independent of the copolymer used or the particle size within 30 min with no significant differences between the nanoparticles and microparticles nor between the PGLA used (50:50; 72:25; 85:15; Figure 8). Continuous increase in uptake was seen within the first 3 h for all formulations used except for the particles studied on the RAW264.7 cell line, which showed a sustained uptake from 30 min to 3 h.

The functional ability of antigen being taken into the cells was tested using DQ-OVA. Thus, DQ-OVA was encapsulated into the PLGA nano- and microparticles in order to evaluate the ability of the formulated PLGA particles to deliver functional OVA to the cells (Figure 8). The results showed that 49–65% of the DQ-OVA delivered from the nanoparticles is degraded by RAW264.7 cells, whilst THP-1 cells showed 72–83% and the MH-S cells performed similarly, with 51–74% DQ-OVA undergoing proteolytic degradation after three hours. On the other hand, when DQ-OVA was entrapped inside PLGA microparticles, very low antigen processing was observed. PLGA microparticles showed below 7% antigen cleaved when exposed to RAW264.7 and THP-1 cells and up to 16% when exposed to MH-S cells. Overall, MH-S cells (alveolar macrophages) showed the best and most rapid uptake for both nano- and microparticles which represent the target place for the delivery of the TB subunit vaccine.

### 3.5. Prime-Pull Vaccination Using CAF01:H56 as Primer and PLGA:H56 as Mucosal Booster

We have previously determined the critical process parameters for the development of dry powder PLGA nanoparticles and microparticles suitable for the delivery of the H56 vaccine candidate to the deep lungs (as determined through a wide range of in vitro techniques). PLGA:H56 nanoparticles were prepared using microfluidics at a TFR 10 mL/min and FRR 1:1 and PLGA:H56 85:15 microparticles using the double emulsion method. The amount of H56 antigen was fixed at 0.25 mg/mL (10 µg per dose) for all the formulations.

#### 3.5.1. Mucosal Boosting with PLGA:H56 Nano- or Microparticles Does not Enhance the Humoral and Cellular Immune Responses

**Figure 9:**
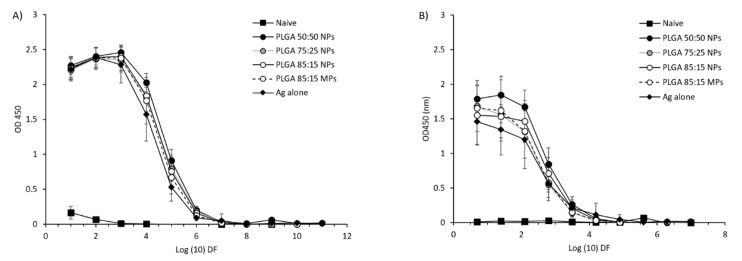
Antigen-specific humoral immune responses in (**A**) serum and (**B**) supernatants from lung lymphocytes after prime-pull immunisation (2 × s.c. CAF01:H56 and 1 × i.n. PLGA:H56). Serum samples from 6 individual mice in each vaccine group were isolated 2 weeks after the booster immunization. H56-specific serum production of immunoglobulin G (IgG) was measured by ELISA. ELISA OD450 values are represented as mean values ± SD, n = 6.

CB6F1 mice were subcutaneously primed with H56 (Ag85B-ESAT6-Rv2660) in combination with the CAF01 adjuvant (which promotes Th1/Th17 responses) and boosted with different PLGA copolymers encapsulating H56 as outlined previously in Figure 1. Only the impact of the format of the mucosal booster was tested based on recent studies demonstrating the combination of prime and boost gave advantages over parenteral priming alone [22,23]. Systemic and mucosal antigen specific IgG responses were measured in blood serum and lung lymphocytes, respectively, 14 days after the last immunisation by direct ELISA. From the results in Figure 9, it can be seen that no significant differences were observed between the vaccinated groups. All the PLGA formulations as well as the mice that received the antigen alone gave significant (*p* < 0.001) higher H56 specific IgG immune responses when compared to the naïve (unvaccinated) group (Figure 9A). H56-specific IgG responses in the supernatants from lung lymphocytes showed similar results (Figure 9B).

**Figure 10:**
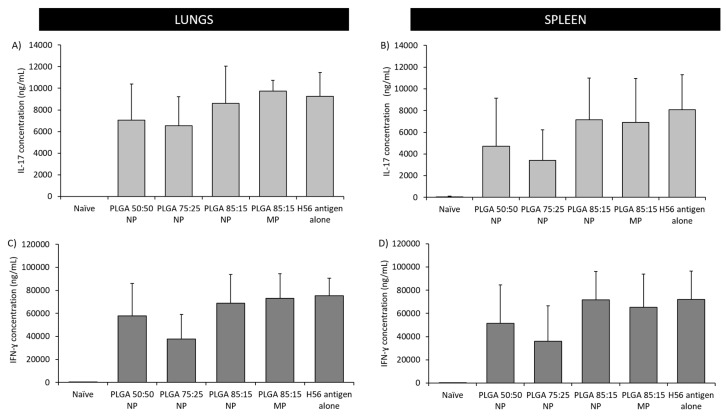
Antigen-specific cellular immune responses in supernatants from re-stimulated splenocytes and lung lymphocytes after prime-pull immunisation (2 × s.c. CAF01:H56 and 1 × i.n. PLGA:H56): IL-17 concentration in the (**A**) lungs and (**B**) spleen respectively; IFN-γ concentration in the (**C**) lungs and (**D**) spleen respectively. Individual spleens and lungs were isolated 2 weeks after the booster immunization. H56-specific cellular production of cytokines was measured by ELISA. Calculated mean concentration values (bars) ± SD are represented.

The production of Th1 and Th17 type immune responses was studied by quantification of the H56-specific IFN-γ and IL-17 cytokines respectively in the supernatants of re-stimulated splenocytes and lung lymphocytes (Figure 10). These cytokines were selected due to their importance in the protection against pulmonary TB [3,24]. Results showed comparable secretion of both cytokines, IL-17 and IFN-γ, in the spleen and lungs. Stimulation was found to be independent of the PLGA booster administrated, since no significant differences between the vaccinated groups were found. All vaccinated groups produced high and comparable cytokine responses, whereas the unvaccinated group did not stimulate the production of any of the cytokines studied (Figure 10).

#### 3.5.2. The Location of T-Cell Responses is Important for Protection against TB: Prime-Pull Immunisation Stimulates Cytokine Production in the Lung Parenchyma

Investigation of the distribution of the CD4+ T cell profiles was studied using a well-established in vivo staining technique described by Anderson et al. [11]. FITC-labelled anti-CD45 monoclonal antibody was intravenously injected 3 min prior to euthanisation in order to differentiate between the cells located in the vasculature and the parenchyma. By this method, blood cells will be stained (positive staining) opposite to the cells located in the parenchyma (negative staining). Therefore, the H56-specific CD4+CD44+ T cell cytokine profile (producing IFN-γ and IL-17) in the individual cells in the lungs, spleen and lymph nodes (mediastinal and traqueobroncheal) were investigated using imaging florescence activated cell sorting (iFACS) and by gating for activated CD4+ T cells. The gating strategy for the flow cytometry is shown in Supplementary Figure S2. The in vivo staining technique is more noticeable for the lungs, as they contain a large number of blood cells whereas the spleen and lymph nodes contain very little. Subsequently, the amount of positive (vasculature) CD4+ T cells in the spleen and lymph nodes was minimum and therefore, non-quantifiable. The CD4+ T cell population in the lungs was differentiated between vasculature (positive staining) and parenchyma (negative staining).

**Figure 11:**
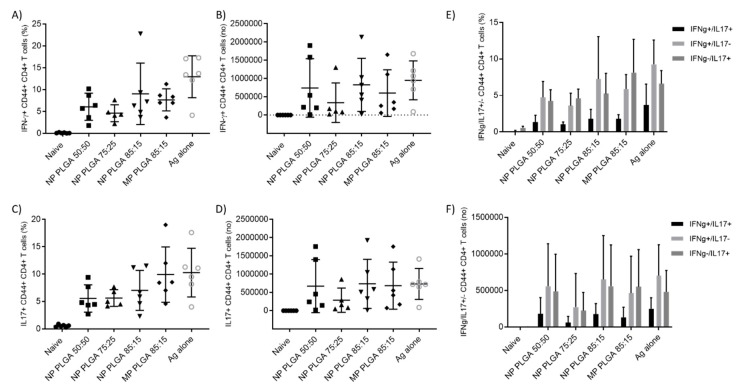
T cell responses following vaccination 2 × s.c. CAF01:H56 and 1 × i.n. PLGA:H56 in CB6F1 mice (n = 6). Two weeks after intranasal boosting with the antigen, the cells from the lungs were stimulated with H56 antigen and cytokine production was assessed by icFACS. (**A**) Percentage and (**B**) number of H56-specific CD4+ T cells producing INF-γ and (**C**) percentage and (**D**) number of H56-specific CD4+ T cells producing IL-17 in the lung parenchyma. Figure (**E**,**F**) show the percentage and number of H56-specific CD4+ T cells producing both INF-γ/IL-17, respectively.

Cells expressing CD44+ were measured for the identification of vaccine memory CD4+ T cells [25]. Analysis of the H56 specific production of IFN+ CD44+ CD4+ T cells and IL-17+ CD44+ CD4+ T cells located in the parenchyma as percentage or number of cells producing these intracellular cytokines is shown in Figure 11. All vaccinated groups produced significantly (*p* < 0.05) higher levels of both IFN-γ and IL-17 in the lung parenchyma compared to the unvaccinated group, independent of particle size and copolymer used. There was no significant difference between the responses stimulated by the various PLGA formulations and no PLGA formulation gave significantly different responses to that of H56 alone. In general, cytokine production in naïve mice was barely detectable and cells in the parenchyma were able to produce more cytokines than cells in the vasculature (data not shown). These results confirm the ability of PLGA to deliver H56 to the lungs with similar levels of antigen-specific cytokine producing CD4+CD44+ T cells observed for all the vaccinated groups. Expression of cytokine combinations (IL-17+/IFN-γ+, IL-17+/IFN-γ- and IL-17-/IFN-γ+) were measured and the majority of the cells were single producers of cytokines, either IL-17 or IFN-γ single-positive CD4+ T cells, were found in the lung parenchyma (Figure 11E,F). Vaccinated groups resulted in a significantly higher (*p* < 0.001) increase in antigen-specific CD4+ T cells compared to control mice (unvaccinated group). Boosting with PLGA:H56 nanoparticles and microparticles induced equivalent percentages of Th1 and Th17 CD4+ T cells as boosting with H56 antigen alone. 

#### 3.5.3. Antigen Specific CD4+ T Cells Responses in the Spleen and Lymph Nodes

**Figure 12:**
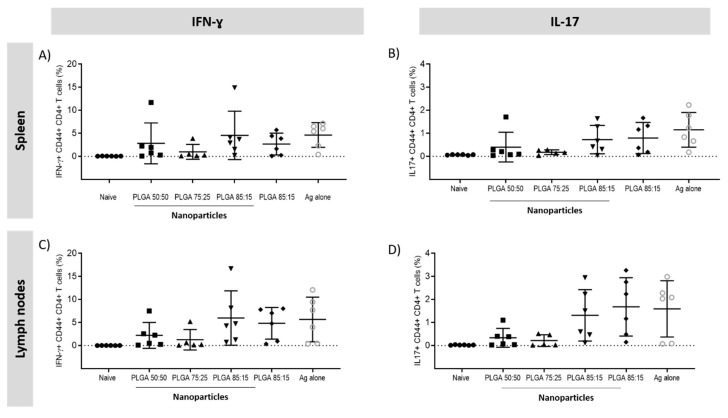
Percentage of IFN+CD4+CD44+ and IL-17+CD4+ CD44+ producing T cells measured by intracellular staining in the splenocytes and lymph nodes of vaccinated mice. T cell responses following vaccination 2 × s.c. CAF01:H56 and 1 × i.n. PLGA:H56 in CB6F1 mice (n = 6). Two weeks after intranasal boosting with the antigen, the cells were stimulated with H56 antigen and cytokine production was assessed by icFACS: Percentage of (**A**) IFN-γ and (**B**) IL-17 in the spleen; percentage of (**C**) IFN-γ and (**D**) IL-17 cytokines in the lymph nodes.

Secretion of IFN-γ and IL-17 responses in the spleen and lymph nodes was assessed. Regarding the CD4+ T cells responses in the spleen and lymph nodes, all the vaccinated groups secreted significantly higher (*p* < 0.001) amounts of cytokines compared to the naïve group (Figure 12). No significant differences were found between the PLGA:H56 booster groups but for the for the F1 mice vaccinated with PLGA:H56 75:25 nanoparticles, which showed a significantly decrease (*p* < 0.05) in the production of IFN-γ+ and IL-17+ CD4+ CD44+ T cells.

## 4. Discussion

One of the main problems limiting the use of PLGA for the development as a delivery system is its physical and chemical instability in aqueous suspensions. After a prolonged period, PLGA particles tend to fuse/aggregate and the payload leaks from the particle due to hydrolytic degradation/erosion [20,26]. Preparation of PLGA delivery systems as dry powders can increase the physical and chemical stability of these particles and allow for the engineering of particles with suitable aerodynamic parameters to achieve deposition in the appropriate lung region. An optimal method to enhance the PLGA particle stability is freeze-drying. However, this method can promote fusion or aggregation of the particles [20,21]. Therefore, cryoprotectants are added prior to freezing to protect and to stabilise the particles, preserving their physical and chemical characteristics and helping with the redispersion of the powder in aqueous media [21,27]. The most common cryoprotectants are sugars, which exert their stabilising effect through the formation of hydrogen bonds with the polar groups at the nanoparticles surface, also known as water replacement theory. Another hypothesis of the sugars mechanism of action is the particle isolation theory, which takes place during the freezing step (above Tg) and whereby sugars avoid aggregation of the nanoparticles by isolating particles in the unfrozen part [28]. Amino acids such as alanine, L-leucine and glycine are also used in the formulations of dry powders working as dispersibility enhancers and improving aerosolisation performance of the powders for inhalation [17,29]. Therefore, in order to screen the effect of the addition of cryoprotectants to the PLGA particles, different concentrations of the sugar sucrose and the amino acid L-leucine were added into the formulations after manufacture (Figure S3). The combination of sucrose (10%) and L-leucine (1%) used here, allows for the stabilisation of the particles during the freeze-drying processes, irrespective of them being nano- or microparticles and the PLGA copolymer used. Previous studies have shown that PLGA nanoparticles could be freeze dried with satisfactory results when sucrose was added at a concentration of 20% [30] and Chacon et al. demonstrated that at least 5% of cryoprotectant is fundamental to preserve the initial particle characteristics [31].

In vitro studies using the NGI (Pharmacopoeia approved airway simulator) demonstrated the complete release of the powder during aerosolisation, which is representative of an adequate dose uniformity. Therefore, no static interaction of the dried powder with the capsule material occurred. FPF was also very high (50–60%), which correlated to the studies by Seville et al., who showed high ED% (approximately 95%) with powders formulated using spray drying (SD) and containing L-leucine [17]. These powders also showed high FPF and FPD (50–85% and 500–700 µg, respectively, depending on the concentration of L-leucine added into the formulation). Several groups have studied the use of L-leucine for the enhancement of the aerosolisation properties of the powders (mainly using SD). For example, Najafabadi et al. [32] demonstrated addition of 9% *w*/*w* L-leucine into a formulation containing 91% sodium cromoglycate increased the dispersibility of the formulation when produced using SD. Chew et al. [33] investigated the use of amino acids (5% *w*/*w*) in combination with sodium cromoglycate for the preparation of SD powders. Their studies showed that the accumulation of L-leucine on the surface of the particles produced better dispersibility when compared to any of the other amino acids studied. Chow et al. [34] showed the improved aerosolisation of naked siRNA using L-leucine as a dispersibility enhancer with high ED (80%) and modest FPF (45%). Other groups reported that the combination of the sugar mannitol and L-leucine produced the highest ED and FPF > 80% compared to other combinations of sugars and amino acids [35]. Eadara et al. used L-leucine for the development of SD powder containing drugs for the treatment of TB and the obtained results showed that the addition of L-leucine increased the FPF up to 70% [36]. Some researchers have hypothesised that the improvement in the aerosolisation of powders containing L-leucine is due to its molecular structure, specifically to the hydrophobic alkyl side chain [17,37]. 

Cellular studies in three macrophage cell lines showed low toxicity for both PLGA nano- and microparticles, which correlates with the results reported in literature (e.g., [38,39]) as the high safety profile of PLGA has been extensively reported (it was approved for human use by the Food and Drug Administration and the European Medicine Agency). PLGA suffers hydrolytic degradation in the body, and degrades into its nontoxic metabolites lactic and glycolic acid. Regarding cellular particle uptake, no major differences were observed in uptake between nano- and microparticles, suggesting that in these studies the particle size is not making any difference. However, antigen processing studies shown differences and a similar trend in the three cell lines tested. The differences observed were attributed to the particle size since PLGA nanoparticles showed much higher antigen degradation compared to the microparticles. This has previously been reported in literature, for example, Akagi et al. showed that PLGA nanoparticles of 40 nm loading DQ-OVA showed less degradation of the protein compared to PLGA nanoparticles of 200 nm in RAW264.7 cells, demonstrating the importance of the particle size for the antigen processing within polymer nanoparticles [40]. The larger size of the microparticles might contribute to a reduced antigen release due to slower polymer degradation in comparison to nanoparticles.

After formulation development and in vitro studies, the immunogenicity of a prime-pull approach by parenteral priming with CAF01:H56 followed by intranasal administration of H56 antigen encapsulated in PLGA as inert delivery system was investigated. Lack of understanding of how TB immunology works in humans has delayed the development of an efficacious and protective vaccine against this infectious disease [41]. The H56 vaccine candidate is a fusion protein from Mtb which is based on early secreted TB antigens Ag85B and ESAT-6 (ESAT-6 remains expressed during chronic infection) and the latency phase secreted antigen Rv2660; therefore, it is considered a multistage subunit vaccine, as it can be employed as a prophylactic and post exposure vaccine [42]. This multistage TB vaccine antigen has demonstrated to be protective in animal models and it is currently in clinical trials (phase 2a). It has been previously reported that the administration of the adjuvant formulation CAF01 in combination with H56 antigen by parenteral route produces early CD4 T cell responses in the lungs after TB infection [5]. Recent studies by Woodworth et al. have also shown that parenteral administration of CAF01:H56 vaccine followed by respiratory mucosal boost with the same subunit vaccine further increases the recruitment of memory T cells to the lung, enhancing protection against TB [43].

When these particles were tested in vivo as part of a prime-pull approach, mice boosted with PLGA:H56 nano- and microparticles gave comparable results to those boosted with H56 antigen alone, and no enhancement of the cellular and humoral immune responses were observed. It is important to find the best particle attributes (particle size, size distribution and morphology) for the delivery systems in order to elicit the adequate immune response. However, this has been challenging, as many studies evaluating the influence of the particle size on evoking immune responses are in disagreement. Studies carried out with PLGA particles with different sizes showed that administration of PLGA microparticles evoked increased IgG immune responses compared to PLGA nanoparticles [44,45], whereas studies by Carcaboso et al. showed that similar IgG responses were obtained after administration of PLGA nanoparticles with sizes of 110 nm and 800–900 nm [46]. Regarding copolymer composition, studies by San Roman et al. on PLGA microparticles encapsulating OVA using copolymers 50:50 and 75:25 showed similar IgG1 responses for both formulations, whereas the IgG2a responses were higher for the more hydrophobic copolymer (75:25) [47]. In this study, four different particle sizes were investigated along with the influence of the PLGA copolymer and the results show that neither size nor polymer choice have an impact, similar to the work of Carcaboso et al. [17]. This is in contrast to the previous in vitro uptake and antigen processing studies which suggested that PLGA nanoparticles were more effective compared to PLGA microparticles. In vitro results showed similar particle uptake for both nano- and microparticles independently of the copolymer used whereas the antigen processing studies in the macrophages cell lines were significantly better for PLGA nanoparticles.

Incorporation of the antigen within the nano- and microparticles was not detrimental to the antigen efficacy, and incorporation of the antigen within these systems allows for both of them to be delivered in a stable dry-powder format. The importance of IgG production has been reported elsewhere, low levels of IgG are associated with higher susceptibility to TB infection, thus, stimulation of early antibody responses at the site of infection might contribute to the fight against the infection by neutralising Mtb antigens [48,49]. There is a wide variety of studies using PLGA particles for vaccine delivery showing that certain vaccine attributes, such as particle size and polymer intrinsic properties, influence the generated immune response. Published data generally agrees that PLGA microparticles produce enhanced humoral immune responses whereas PLGA nanoparticles generate higher cellular responses [44,50]. For example, Gutierro et al. showed that PLGA microparticles (~1 µm) loading bovine serum albumin (BSA) as a model antigen, produced higher IgG serum antibody titres than the BSA:PLGA nanoparticles (200 and 400 nm) after any of the three administration routes evaluated: subcutaneous, intranasal and oral [44]. Regarding polymer properties, San Roman et al. demonstrated that PLGA 75:25 microparticles encapsulating OVA produced stronger IFN-γ responses compared to PLGA 50:50 when administered intradermally [47]. This result was attributed to the hydrophobicity of the copolymer, since it is likely that the interaction of the copolymer 75:25 with the APCs resulted in superior T cell activation [47]. Moreover, Thomas et al. developed vaccine delivery systems for a hepatitis B antigen (HBsAg) based on PLGA 85:15 and 50:50 nanoparticles (~770 and 470 nm respectively). He showed that HbsAg:PLGA 85:15 nanoparticles produced higher humoral and cellular immune responses compared to the other copolymer after pulmonary administration [51]. These immunological differences were attributed to the hydrophobicity of the copolymer 85:15 and its particle size [51]. However, studies on cationic PLGA 50:50 and 75:25 microparticles absorbing HBsAg showed similar humoral (IgG) and cellular responses (IFN-γ and IL-2) for both copolymers after subcutaneous vaccination [52]. Our results showed no differences between the nano- and microparticles, nor the copolymer used.

Understanding the participation of the cells during the production of immune responses is crucial for vaccine development. Production of antigen specific CD4+ T cells and the homing of these cells to the lungs is of vital importance to fight against Mtb infection [53]. Their distribution is important since the cells located in the lung parenchyma and, therefore, in direct contact with the pathogen will be able to protect against the infection [54]. The enhancement of mucosal CD4+ T cells producing IL-17 is important for the development of a protective vaccine against TB as well as the production of IFN-γ CD4+ T cells, which have been previously demonstrated to be of high importance against TB in humans [55,56]. The current TB vaccine, BCG, does not stimulate T cells in the lungs, which has been attributed to its lack of efficacy in protecting against pulmonary TB. Studies carried out in guinea pigs showed that after mucosal vaccination with BCG, better protection against TB was found when compared to the conventional intradermal route [57]. Recent studies have shown the increased local immune responses obtained by mucosal vaccination and consequently, improved protection against TB [56]. The importance of the recruitment of T cells to the lungs has recently been demonstrated for protection against TB infection. The location of these T cells is crucial for the efficacy of the vaccine since the T cells have to be in direct contact with the Mtb infected macrophages [4,5,55]. Therefore, respiratory mucosal vaccination is believed to be the best way to teach the lung macrophages and dendritic cells (DCs) how to fight Mtb infection and to recruit antigen specific T cells to the site of infection (lung parenchyma and airway) [6,58].

This work demonstrates the design of a TB subunit vaccine containing the TB vaccine candidate H56 (Ag85B-ESAT6-Rv2660) as part of a prime-pull protocol where the Th1/Th17 promoting adjuvant CAF01 was administered by parenteral priming followed by a respiratory mucosal booster with PLGA:H56. Results from this study demonstrate that PLGA:H56 nanoparticles and microparticles can be produced in a dry powder format which can work as effectively as the administration of free antigen. This is key, as it is unlikely to find an antigen that after administration alone produces these high immune responses as generally, administration of free antigen is poorly immunogenic. The H56 vaccine candidate could be encapsulated within a well-characterised delivery system without losing its immunogenicity in a murine model. Stimulation of the adequate cell subset population in the right location and in the right quantity is believed to be a critical factor for controlling Mtb growth. Studies by Woodworth et al. [43], following a protocol based on 3 × s.c. administrations of CAF01:H56 and 2 × s.c. followed by 1 × i.n. CAF01 in CB6F1 mice showed an improvement in the recruitment of the T cells to the lungs (parenchyma) after mucosal vaccination compared to the subcutaneous route. Regardless of the increased antigen specific responses in the lung parenchyma, the narrowed protection showed after challenge with Mtb demonstrates the limited power of the vaccine-induced T cells to regulate Mtb infection [43]. A more recent study, showed no enhancement in the protection against aerosol Mtb challenge despite the T cell population generated and the accelerated T cell response in the lungs [22]. Furthermore, another study by Asshurst et al. for pulmonary administration of PLGA encapsulating a TB antigen also demonstrated that although the T cell immune responses were increased in the lungs, these did not enhance the protection against TB after challenge [59]. These data suggest practical limitations on the ability of vaccine-elicited T cells to control aerosol Mtb infection.

## 5. Conclusions

Alveolar macrophages can be found in multiple locations including the vasculature and the parenchyma and represent the first line of defence against Mtb. The importance of the recruitment of T cells to the lungs for protection against TB infection has recently been demonstrated. The location of these T cells is crucial for the efficacy of the vaccine since the T cells have to be in direct contact with the Mtb infected macrophages. Therefore, respiratory mucosal vaccination is believed to be the best way to teach the lung macrophages and DCs how to fight Mtb infection and to recruit antigen specific T cells to the site of infection. Here, we evaluated a prime-pull immunisation approach for a vaccine against pulmonary TB, consisting of priming with CAF01:H56 followed by a mucosal boost with PLGA:H56. PLGA encapsulating the TB vaccine candidate H56 was formulated in a scale independent manner and lyophilised to a dry powder format for aerolisation into the lungs. Results shown here demonstrate the applicability of this manufacturing method to deliver encapsulated antigen to the lungs via the intranasal route of administration. The immune responses generated by this approach indicate the retention of the immunogenicity of the antigen encapsulated within a lyophilised PLGA delivery system. Although no enhancing effect could be observed compared to the administration of antigen alone as a boost, the work here could provide the foundations for the scale independent manufacture of polymer based delivery systems encapsulating antigens for inhalation/aerolisation delivery to the lung mucosa. Moreover, the exclusion of the cold-chain requirement would contribute with the reduction of the vaccine cost and it would facilitate the stability of the vaccine during transit and storage.

## Supplementary Materials

The following are available online at https://www.mdpi.com/2076-393X/8/1/10/s1, Figure S1: Schematic representation of the manufacturing processes for the PLGA nanoparticles and microparticles encapsulating H56 antigen, Figure S2: Gating strategy for identifying activated lung-resident CD4+ T cells, Figure S3: Overview of the effect of the choice of cryoprotectant into the PLGA 50:50, 75:25 and 85:15 nanoparticles manufactures using microfluidics.
