# Supplementary material for: Investigating Prime-Pull Vaccination through a Combination of Parenteral Vaccination and Intranasal Boosting
Source: Vaccines (Basel). 2019 Dec 31;8(1):10. doi: 10.3390/vaccines8010010 (PMC7157738; doi:10.3390/vaccines8010010)
Supplement: Supplementary file 1 [file vaccines-08-00010-s001.pdf]

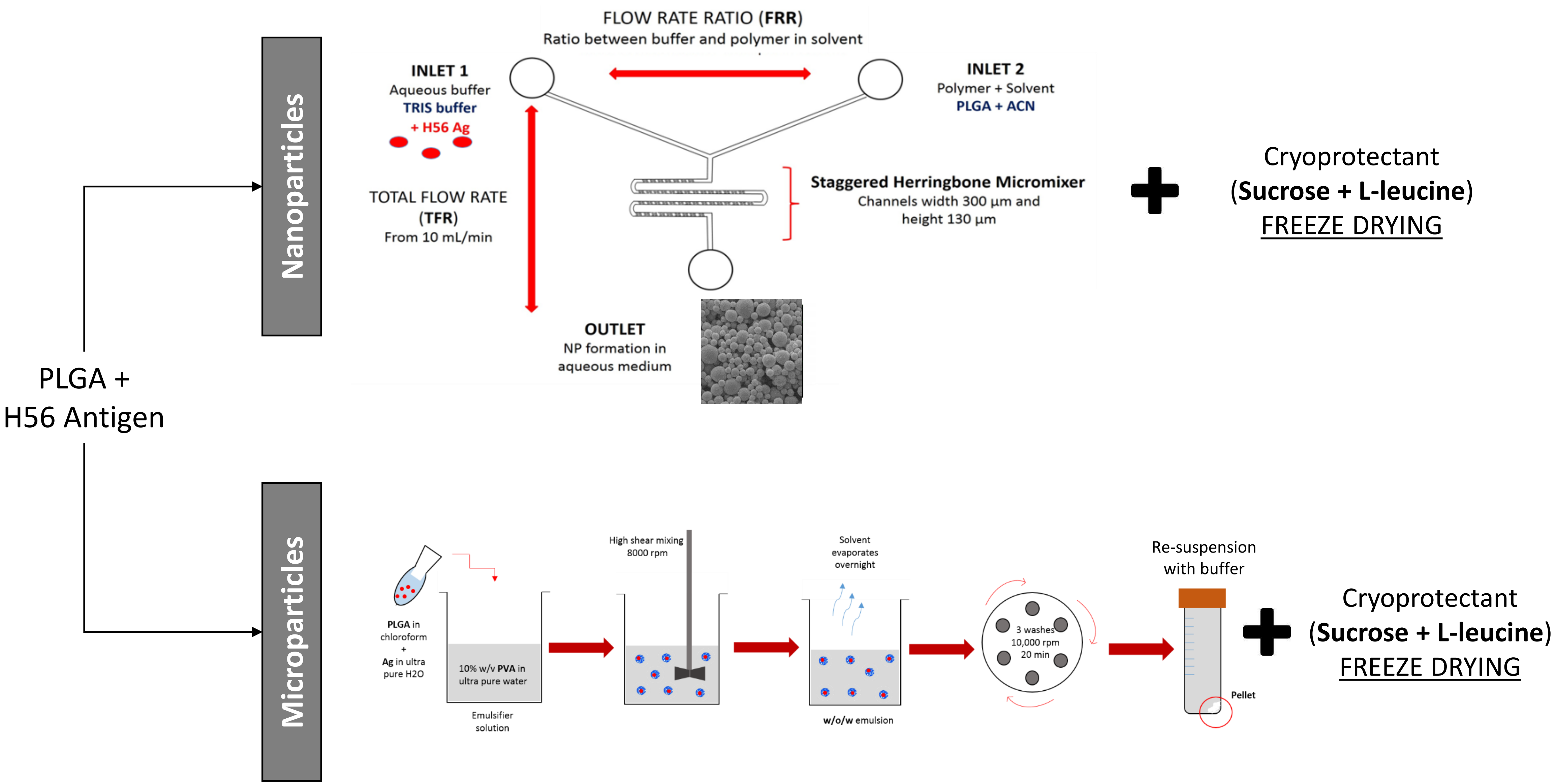

Figure S1. Schematic representation of the manufacturing processes for the PLGA nanoparticles and microparticles encapsulating H56 antigen. Nanoparticles were produced using microfluidics at a TFR 10 mL/min and FRR 1:1 whereas microparticles were prepared using the double emulsion method. Cryoproctants were added post-manufacture and then, particles were freeze dried.

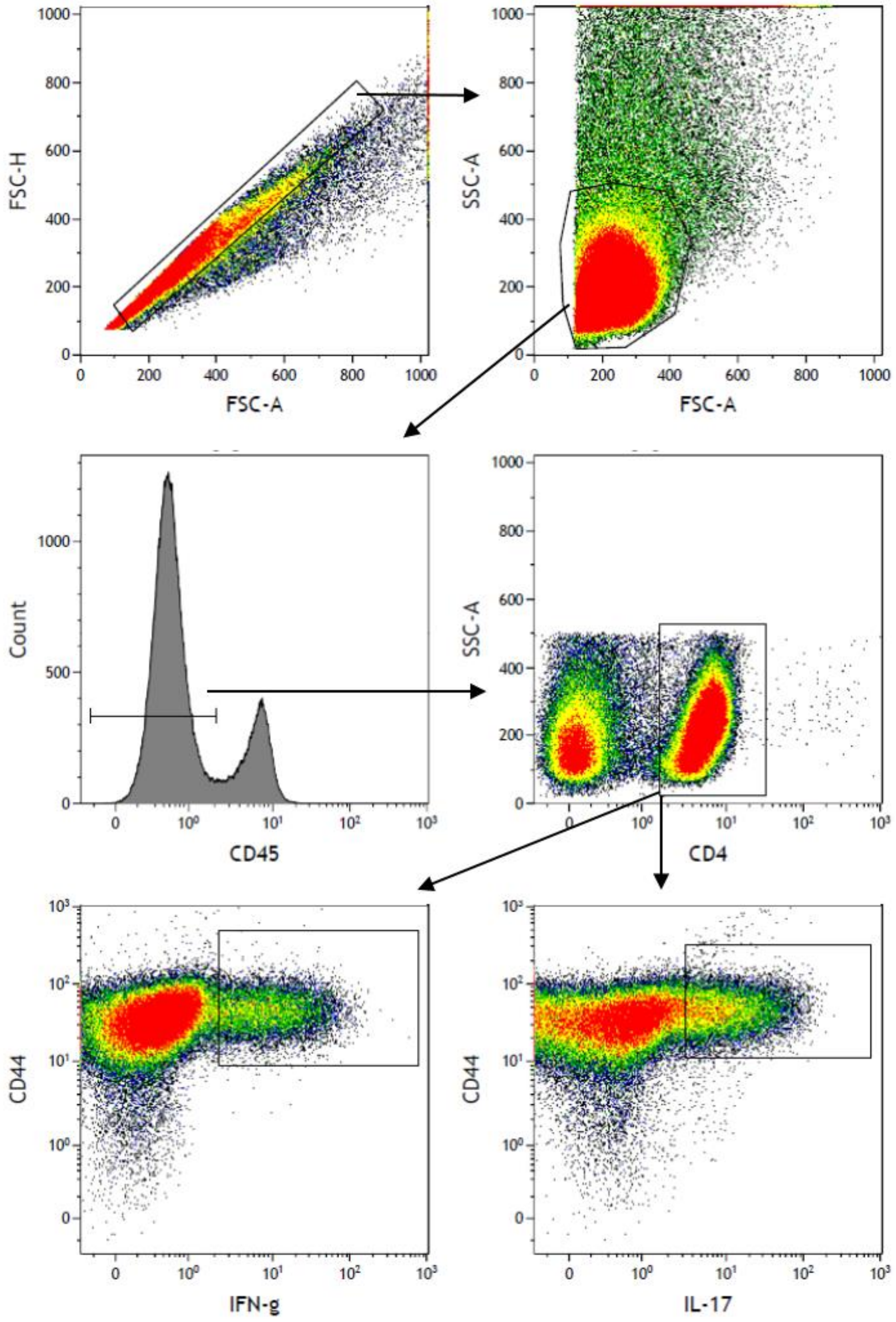

Figure S2. Gating strategy for identifying activated lung-resident CD4$^+$ T cells. First singlets and then lymphocytes are identified, from which lung-resident cells are characterized as CD45neg cells. Within the CD4$^+$ population, CD44$^+$, IFN-γ$^+$ and CD44$^+$, IL-17$^+$ cells are identified. Double-positive cell populations are identified with Boolean gating. This sample from a representative mouse which received PLGA 85:15 nanoparticles.

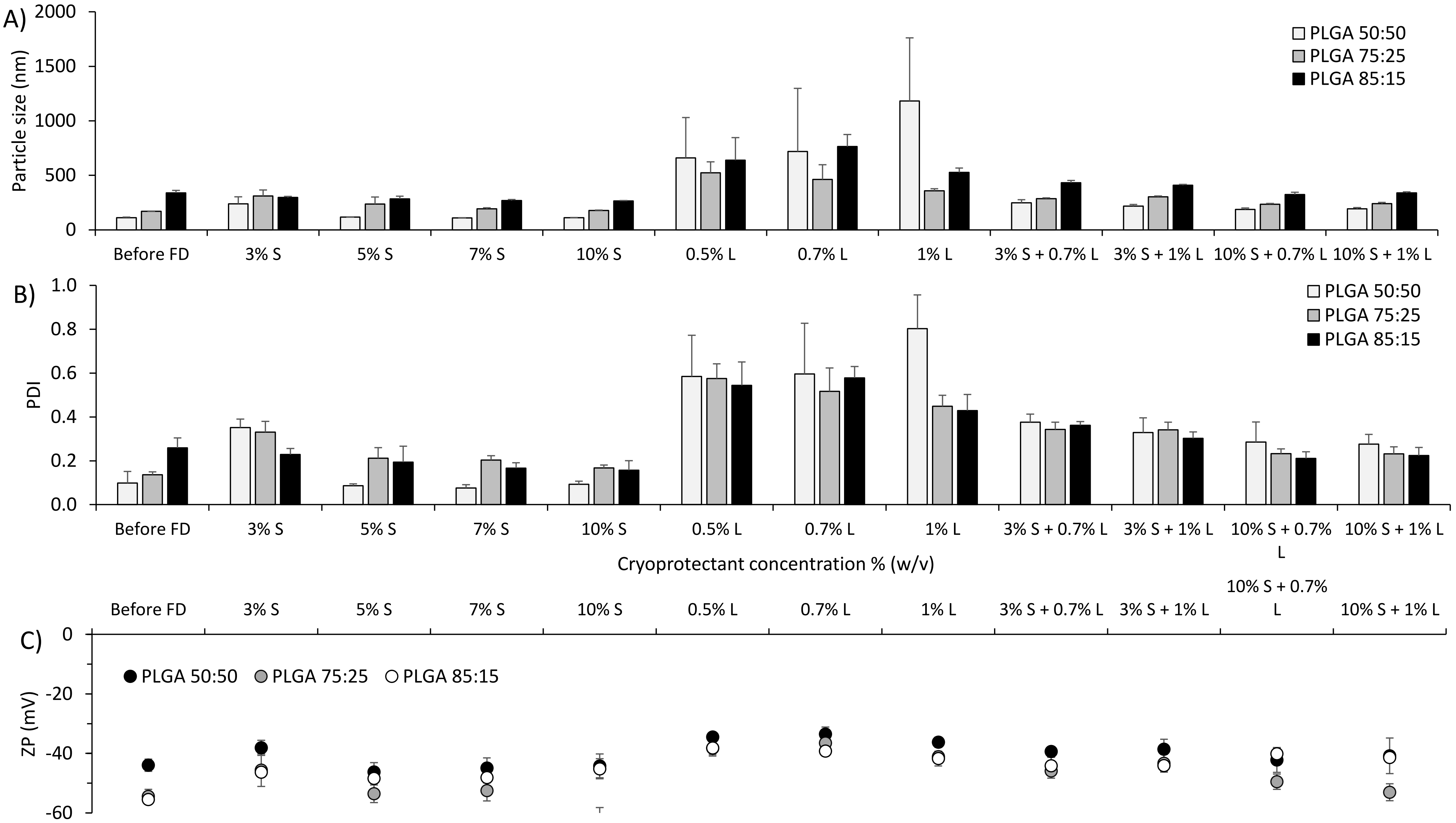

Figure S3. Overview of the effect of the choice of cryoprotectant into the PLGA 50:50, 75:25 and 85:15 nanoparticles manufactures using microfluidics. Different amounts of sucrose (3, 5, 7 and 10% w/v), L-leucine (0.5, 0.7 and 1% w/v) and a combination of both (3% sucrose + 0.7% or 1% L-leucine; 10% sucrose + 0.7% or 1% L-leucine) were added after manufacture and prior freeze drying. Results show the (A) particle size, (B) PDI and (C) zeta potential of the particles after re-suspension with ultrapure water. Results represent n=3 of three independent batches.
